# Experimental Case Studies about Uniplanar SHS Joints with Full-Overlapped Top Connection

**DOI:** 10.3390/ma15124089

**Published:** 2022-06-09

**Authors:** Patrick Heinemann, Dorina-Nicolina Isopescu

**Affiliations:** Department of Civil and Industrial Engineering, Faculty of Civil Engineering and Building Services, “Gheorghe Asachi” Technical University of Iasi, 1, Prof. Dr. Docent Dimitrie Mangeron Blvd., No. 59A, 700050 Iasi, Romania

**Keywords:** hollow sections, numerical simulation, welding line, construction, steel

## Abstract

Squared hollow steel profiles are commonly used in the construction of offshore structures or building facades. By welding two or more pipes, typical joints are created that are specific for different areas of applications. These joints are less resistant than straight pipes due to the geometrical heterogeneity and the complex stress behavior of the welding. Standards define these joints, but there are restrictions imposed regarding the material or geometry. This paper focused on full-overlapped joints with squared hollow section profiles and on-top connection, which are disregarded in current standards. The aim was to figure out the influence of the inclination angle on the resistance of the joint. In the analysis, experimental and numerical studies were performed. Four different inclination angles commonly used on construction sites were the focus. It was discovered that there is a total diminishment of 46% in the load bearing capacity between the steepest and the most obtuse angles. The structural behavior is non-linear and is influenced by the value of the angles. The second aspect is related to the influence of the steel profile, which is evaluated by a comparison between a squared profile and two circular profiles. It was discovered that the joint made with squared profiles has a higher bearing capacity than the one made with circular profiles, a statement valid for similar thicknesses of the elements.

## 1. Introduction

Squared hollow section (SHS) profiles are commonly used in the construction of offshore structures or for facades of buildings. These profiles are characterized by high resistance to torsion and less intense buckling effects. In some fields of application, it is necessary to connect two or more members to obtain a two-dimensional structure. This joint can be designed by welding or cast steel. Thus, different geometries of the node can be used. Depending on the cross section of the pipes, there are variations including intermediate plates or directly connected pipes. This paper is about welded joints without intermediate plates. Due to geometric discontinuity and complexity in stress distribution, tubular joints require more attention [1]. Current standards, such as Eurocode 3, part 1–8 [2], or design guides, such as the CIDECT [3], define the joints made of hollow section profiles in general. The joints defined by design codes are only valid for regular cases and do not include special variations in geometrical features. The regular case consists of hollow section members, which are connected to the side flank of another member. The load is always set to the inclined member, not to the basic member in the case of a Y- or K-joint. The standards do not include variations related to the steel joint, which are analyzed in this paper. The steel joint is defined as a uniplanar SHS joint with a full-overlapped top connection, commonly used for industrial applications. Two pipes (typically named braces or branches) are full-overlap connected to the end of a vertical member (also called a chord). Beside the geometry, the load scenario offsets the standard. The branches are connected at an inclination angle. The node is the weakest part of the structure due to the geometrical heterogeneity. Researchers’ attention, so far, has generally focused on hollow section joints. Thus, available data concerning this special kind of steel joint are rare, which validates the relevance of the topic. 

Heinemann et al. studied the multiplanar [4] and uniplanar joints [5,6,7]. The joints studied so far mostly include intermediate plates [8], thus the analytical and numerical models are not comparable to the results of tests concerning the specimens made with a full-overlapped welding joint. With the implementation of a steep plate at the end of the chord, the force effect on the branches is concentrated in the center of the steel plate. Depending on the inclination angle, a large deformation may develop at the level of the steel plate if the thickness of the steel plate is too small. Overall, there is a pronounced difference in stress distribution patterns of specimens made with intermediate plates and the ones connected directly. Podkoritovs et al. [9] performed numerical and experimental studies concerning three-dimensional truss joints made of squared hollow section joints. The joint is comparable to the standard defined multiplanar K-joint. The inclination angle is equal to all braces and was set as 43° and, based on the results, it was concluded that, by using a shell numerical model, the outcomes of the numerical and experimental analysis correlated by a factor of 3.9%. Talabani et al. [10] performed numerical studies concerning T-joints with squared hollow section profiles. Different meshing techniques were analyzed to figure out the optimum meshing that should be implemented for the welding line. Based on the numerical results, it was concluded that the welding line should be modelled independently and a refined mesh of 1 mm should be used. Radic et al. [11] and Kalac et al. [12] performed numerical analysis regarding steel truss joints. The cross-section geometries, which are part of standard defined SHS K-joints, were variated. The inclination angle was set to 40.6°. The braces with a smaller width were connected to the side flank of the larger chord. It was concluded that the current design standards do not provide sufficient information regarding the dimensions and the geometrical conditions. Dawod et al. [13] and Soni et al. [14] studied the columns with squared hollow profiles subjected to compression loads. Different geometrical properties were variated. The measurement equipment was similar to the equipment presented in this study. It was discovered that the normalized elastic load capacity of an SHS is reduced significantly if the width-to-thickness (B/t) ratio increases due to the failure transition from flange yielding to flange local buckling [13]. This occurs in the case of slender and short SHS columns. 

The aim of the study presented in this paper is to quantify the influence of the inclination angle on the resistance of the joint. The numerical results are validated by experimental studies. By choosing the right inclination angle the structure can be optimized economically, which is important to the designing engineer. In the second step, the results are compared with two different profile types belonging to the same category of circular hollow sections (CHSs). Thus, it is demonstrated that these types of structures can be optimized by the material and the structural shape choice.

## 2. Materials and Methods

**Figure 1:**
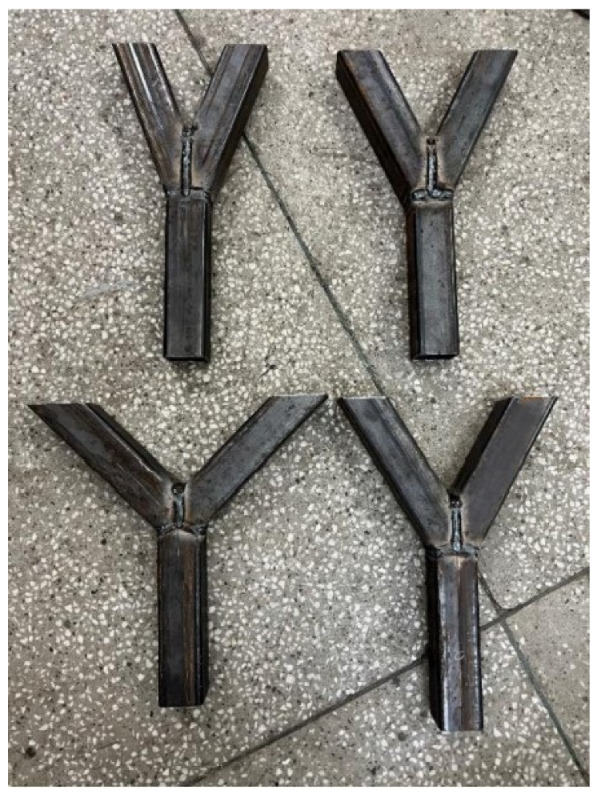
Specimens with different inclination angles.

The specimens are made of squared hollow section profiles with a cross section (QRO) of 50.0 × 2.8 mm, defined by the EN 10219-2 standard [15]. The cross section is equal to all members of the joint. Heinemann et al. discovered that the choice of material has a large influence on the resistance of joints [16]. Younise et al. [17] figured out the importance of the material setup in the case of steel joints. So, in this study, an equal material is set for all specimens. The steel material is defined by its yield stress of 250 MPa, density of 78.5 kN/m^3^ and Poisson’s ratio of 0.3. Konjatic [18] evaluated the influence of welds including the heterogeneity of the material. It was concluded that only repaired welds can significantly influence the resistance of the welding line, and a homogeneous material could be considered in the initial stage. The study outlined in this article is focused on fillet welds made with homogeneous material. Three pipes are used for each specimen. The length of each member is equal and is given as 200 mm. Due to the geometry of the testing machine, the ends of the braces are inclined. Four different inclination angles are analyzed, 20°, 25°, 30° and 45°. The pipes are cut by half of the inclination angle and welded into a Y-shape. Depending on the inclination angle, the length of the vertical welding line varies. The welding line is neglected in some numerical studies regarding steel joints [19]. However, regarding the numerical and experimental analysis in this paper, a 3 mm fillet welding line is used, which has a three-dimensional shape and connects the braces to the chord and the braces itself. To manufacture this welding line, the electric arc welding method is used. The welding line is performed by an attested welder, not by robotics, and the quality of fabrication is proofed by non-destructive tests. Figure 1 illustrates the specimens, including the four different inclination angles. To validate the experimental tests, every test is repeated two times. Overall, the number of tests is given by: 4 (inclination angles) × 3 (verification) = 12 tests.

The evaluation and computation of the cracks is a complex procedure due to different stress states. Multiaxial stress distributions arise in steel joints [20,21,22,23]. The experimental tests involved an analysis addressing whether cracks will appear in the pipe surface or in the welding line. If cracks appear, there will be an adaptation of the numerical model. The analysis in this paper was carried out first up to the elastic limit, then up to the end of the plastic limit state. The force was constantly increased up to the yield limit of the S 235 steel. Before the tests were performed, non-destructive evaluations were carried out to ensure the quality of the welding. Several tests regarding liquid penetration and ultrasonic-based methods were executed. Defects can arise due to the welding process. If defects are detected, the specimen is to be considered inadequate for test and removed.

**Figure 2:**
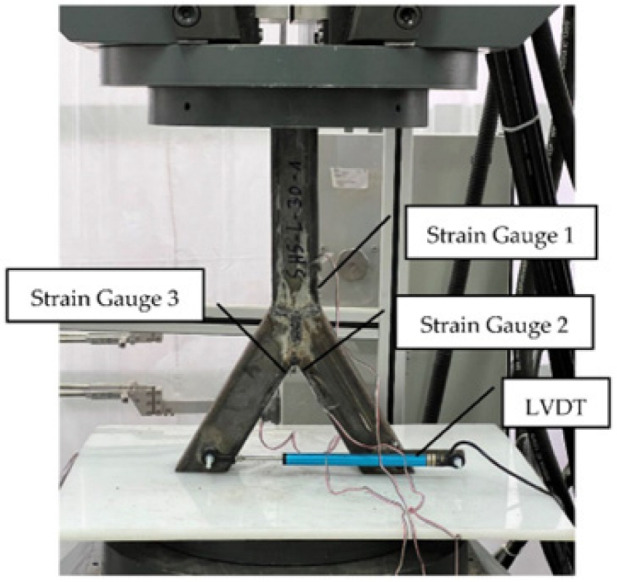
Testing setup.

To measure the stresses indirectly, multiple strain gauges were glued to the specimens in pre-marked positions where the maximum values were expected (Figure 2). The 8 mm one-direction strain gauges were used. These sensors were attached close to the welding line. Due to the curved shape of the weld, the sensors could not be set on the weld. A Zwick/Roel [24] compression force machine was used, with a total capacity of 1000 kN. The tests were performed in the force-control mode at a maximum speed of 10 kN/min. The chord was subjected to an axial compression force. Due to the beveled ends of the braces and the vertical load orientation, two components of the load acted on the specimen. There was, on the one hand, the axial force and, on the other hand, the bending induced in the node. The boundary conditions were set as simple supported. The specimen was laid down on a Teflon [25] plate with a thickness of 10 mm. By compressing the structure, the braces’ ends were pressed into the Teflon material, which resulted in blocked horizontal deflections. Beside this, the material compensated for small imperfections. Figure 2 shows the experimental setup. The vertical force and the deflection were recorded directly with the appropriate machine software.

## 3. Results

### 3.1. Maximum Compression Force SHS

**Figure 3:**
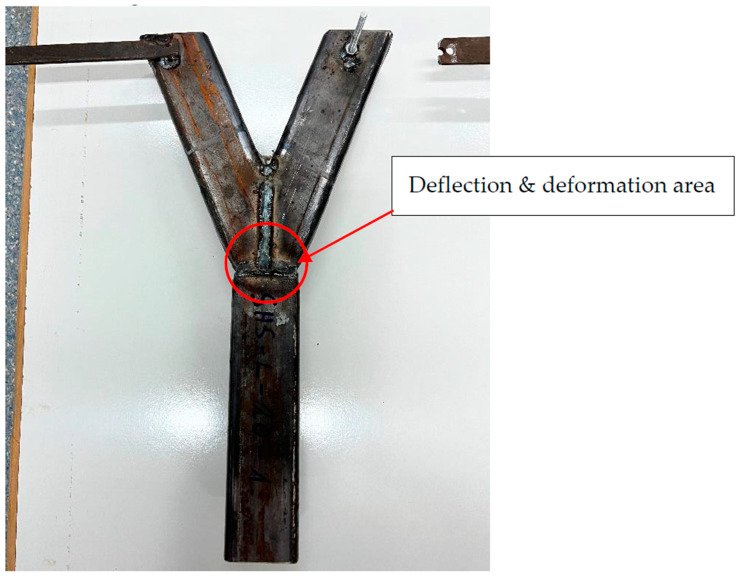
Deformed specimen.

After the experimental tests were finished, no cracks were detected in any specimen. Regarding the deformation shape, wall shear failure occurred in the area of the node. The flat areas of the profile did deform by veiling. Global buckling did not arise due to the small-scale size of the joint. The highest stresses were identified near the edges of the specimen. Figure 3 illustrates the specimen after the test process. The deformation and deflections were small due to the reduced scale of the model. The maximum values arose in the area of the welding line, close to the edges.

**Figure 4:**
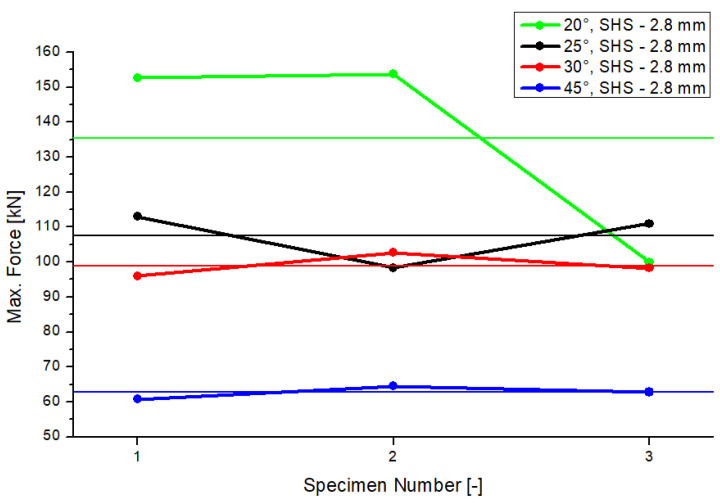
Results of the maximum compression force.

The tests were repeated two times to verify the results of each inclination angle. Figure 4 shows the results of the maximum compression force of the experimental tests. The horizontal lines represent the average of the results according to the colors used for the inclination system (20°, 25°, 30° and 45°). The differences in the results, per model, were rather small. An exception occurred for the 20° model. In this case, the differences between experimental measurements were larger, compared to the other models. A reason for this could be a possible defect in the material in the specimen (no. 3). There is a correlation between the maximum load and the total resistance of the joint. A second conclusion can be drawn from the figure. If there is an increase in the inclination angle, the resistance of the joint will decrease. The differences regarding the average values of the resistance of the 25° and 30° models were small, with a delta of 9 kN. 

**Figure 5:**
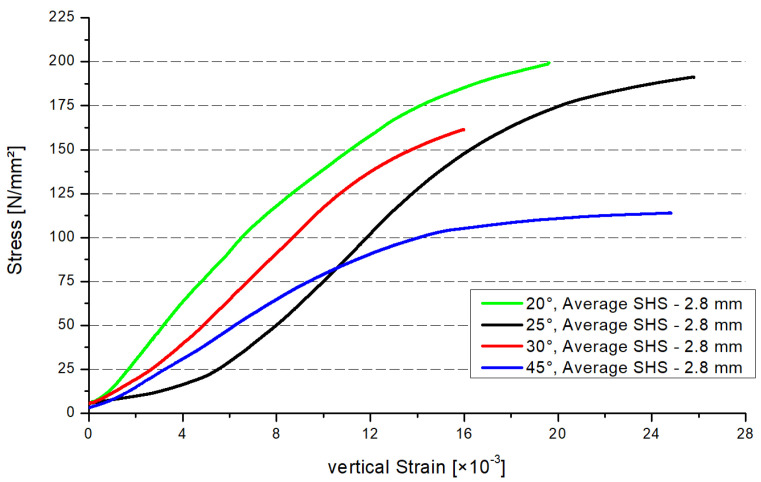
Stress–strain distributions.

Figure 5 shows the stress–strain distribution of the four inclination angle models. Three specimens were tested for each configuration. The stress–strain distribution was estimated for each specimen. In Figure 5, each graph represents the average results obtained for the series. The global stresses were analyzed, being calculated as the maximum force divided by the cross-section area of the profile. By analyzing the maximum stresses, the same conclusion as for Figure 4 can be drawn. The maximum resistance will decrease if the inclination angle increases (Figure 5). There is a gap between each of the curves, except the smaller stresses regarding the 45° model. In the case of the small stresses, the resistance of the 45° model is on average higher than the 25° model. If the stresses exceed the value of 84 N/mm² (intersection point 25°/45°), the resistance of the 25° model is higher than the 45° model. 

### 3.2. Comparison between SHS and CHS

**Figure 6:**
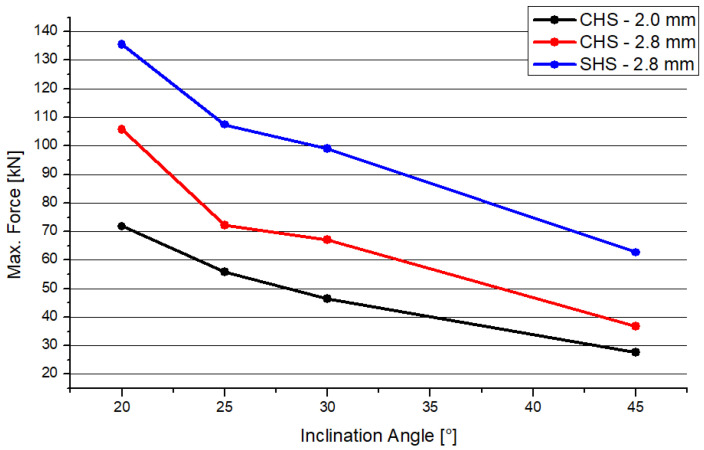
Comparison of the maximum compression force.

Heinemann et al. performed numerical studies concerning uniplanar full-overlapped joints with a top connection, combined with circular hollow section joints (CHS) [26]. The model is comparable to the SHS profile analyzed in this paper. The dimensions of the cross sections (RO 48.0 × 2 mm and RO 48.0 × 2.8 mm [9]) and the analysis process are similar to the SHS model. So, there is one circular profile with the same thickness as the squared profile. The other circular profile is thinner, having a delta of 0.8 mm. The deformation shape is different regarding the two distinct profile joints. In case of the circular profiles, there is no constriction at the side flank of the node. The front side of the joint deforms similar to both joint types. Figure 6 shows the results of the maximum compression force depending on the inclination angle. In accordance with Section 3.1, the resistance will become smaller if there is an increasing inclination angle in the case of SHS. This behavior is represented by the blue curve in the figure. The results of both CHS models are similar to the results of the SHS. There is a decreasing trend. The graphs are nearly superimposable, including a gap of 20 kN or 30 kN, depending on the system. However, both CHS models have a lower maximal force, while the SHS model has the highest resistance when subjected to compression. 

## 4. Discussion

Numerical models were created to determine an analytical form for design engineers. The overall resistance was calculated and verified by comparing the results of the experimental tests.

**Figure 7:**
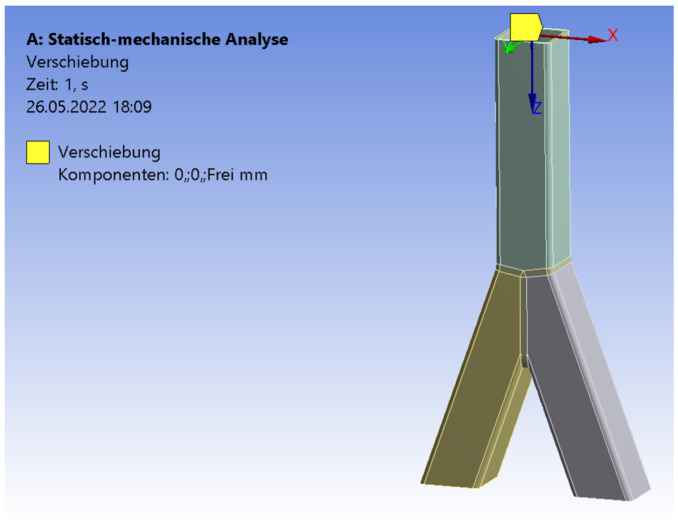
Boundary conditions. Numerical model.

**Figure 8:**
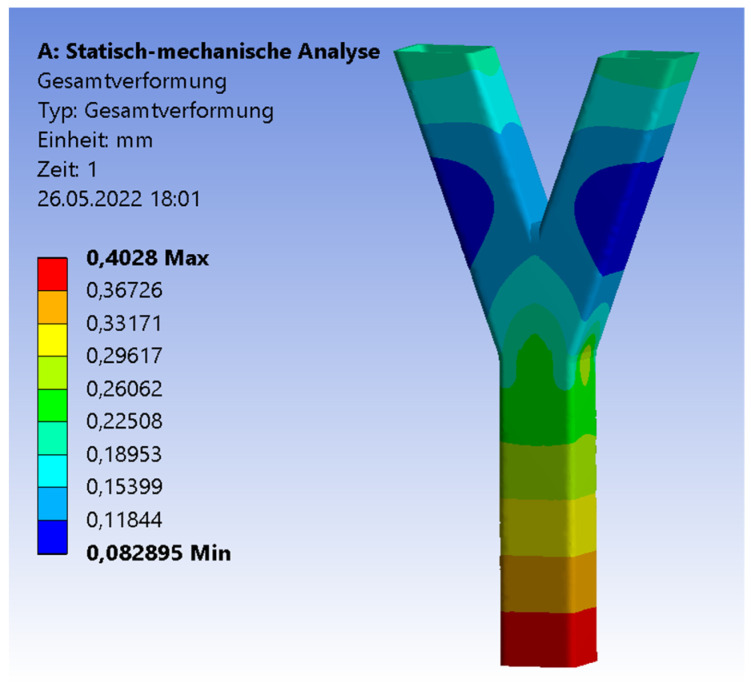
Total deformation for 20° model.

**Figure 9:**
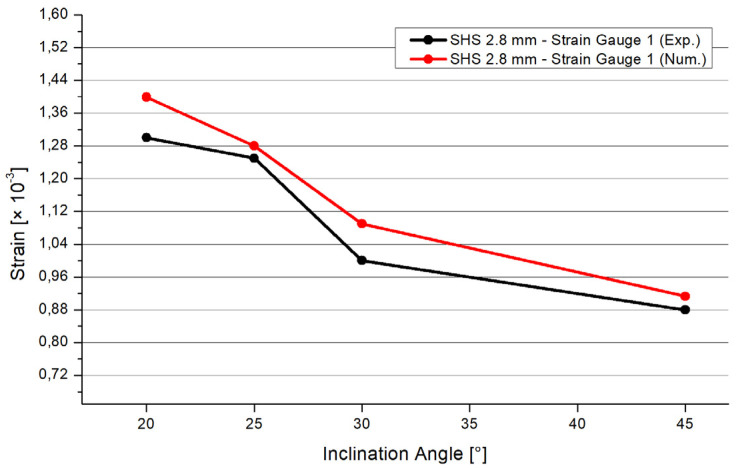
Comparison between the numerical and experimental results. Position 1.

**Figure 10:**
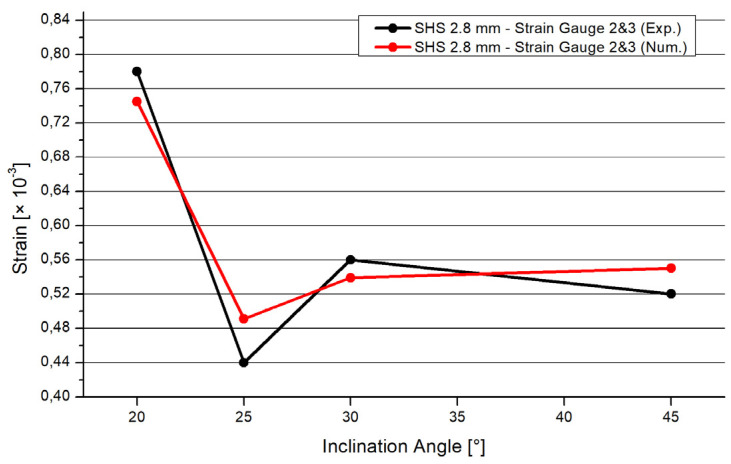
Comparison between the numerical and experimental results. Positions 2 and 3.

The software Ansys [27] was used for the numerical simulations. The three pipes were connected by a three-dimensional 3 mm fillet weld including a triangular shape. To archive a realistic adoption, the connections of the pipes were numerically set as frictionless. The three pipes could deform independently. However, only the welding line transferred the force. The ends of the braces were set as simple supported. The boundary conditions of the chord’s end were set as simple supported, with an unblocked vertical deflection in the z-direction (Figure 7). The end of the chord was subjected to an axial compression force. The realistic behavior of the chords’ ends was in between fixed and simple supported [28]. The value of the compression force was set as equal to the result of the experimental test. The material was equal for the numerical and experimental analyses. An elastic material model was used. The density was 7850 kg/m^3^; the Young’s modulus was 2.1 × 10^5^ MPa. The yield strength was set as 235 MPa, the Poisson’s ratio was 0.3. The mesh was set as 10 mm tetrahedral elements with a quadratic element shape function. In the area of the welding line, there was a refinement of 1.3 mm solid elements to save computational time and to obtain precise results at the same time. To figure out the quality of the mesh, a mesh metric evaluation was carried out. The following values are given as an example in case of the SHS 25° model. The skewness tended to zero with a mean value of 0.28. The Jacobian mean value was 1.03. The mean value of the element quality was 0.79. The convergence was limited to 0.7%. The aspect ratio had max value of 177; the limit factor was 1000. The mean value of the aspect ratio was 1.98. Figure 8 illustrates the distribution of the total deformation in case of the 20° model. The shape fits to the deformation shape of the tested specimens (Figure 3). Figure 9 and Figure 10 show the results of the maximal strain at three different positions in the case of the four inclination angle models. Strain gauge 1 was set to the outer flank of the chord, near the edge of the profile. Strain gauges 2 and 3 were set in the inner flank in between both braces, near the edges of the squared profile. The geometry of the specimen was symmetrical, so the results of strain gauges 2 and 3 are superimposable. If the inclination angle increases, there is a decreasing trend concerning the strains. The results of the experimental and numerical analyses are in a good agreement. The difference is in the range of 7% to 10%. However, it is common to have a discrepancy between the results of laboratory tests and numerical simulations. This is due to the fact that the imperfections related to the manual welding carried out by construction-site workers cannot be considered in the numerical results. Instead, by computing average values of the experimental tests, these effects can be significantly minimalized. Moreover, these small differences in numerical and experimental results can be taken into account and used when a correlation factor is proposed for the analytical models.

By comparing the results to models with intermediate steel plates [5,6,7,8], a higher resistance is achieved concerning the uniplanar full-overlapped joints with a direct pipe connection. The lines of application merge together in the center of the intermediate plate. Especially in case of steeper inclination angles, the force is concentrated in the middle of the plate. Due to this, higher stresses and deformations arise in the steel plate. The steel plate is the weakest part of the structure. In comparison to the directly connected joint, the force is transferred through the cross section. Because of this effect, a higher resistance is achieved. 

The joints fail before cracks appear. The implementation of the crack stress distributions [20,21,22,23] is necessary for the fatigue analysis regarding the full-overlapped steel joint. 

By comparing similar models with circular steel hollow section profiles [26], it can be seen that the squared profile joint has a higher resistance. The behavior of the different inclination angle models is equal in the CHS and SHS cases. As presented in Section 3.2, the squared profile generates a higher stiffness than the circular profile if the cross-section area is comparable. 

Podkoritovs et al. [9] carried out numerical studies concerning three-dimensional truss joints made of squared hollow section joints. As part of a truss, the braces were either in compression or tension. The braces were connected to the side flank of the chord. Due to this, the models are only partly comparable. Shell elements were used in the case of the numerical model. The welding line was not modelled. The distribution of the stresses around the brace-to-chord connection was comparable to the results in this paper. In the case of the full-overlapped joint with an on-top connection, more precise results were achieved by using solid elements. In particular, the welding, where the maximum stresses arise, was analyzed in detail. 

Talabani et al. [10] performed numerical studies concerning different meshing techniques in the case of T-joints with squared hollow section profiles. The advantages of the slice models were explained. However, the computational time increased rapidly and exponentially because the freedom degrees increased. In the case of the study presented in this paper, the meshing method was not appropriate. A linear material was implemented. The slice method has a higher precision concerning non-linear materials regarding the calculation of dynamic loads. 

Radic et al. [11] and Kalac et al. [12] performed numerical analyses of standard defined K-joints. As explained concerning the model in this paper, the design codes had large limits regarding the geometrical shapes of joints. Radic neglected the welding line and focused on joint resistance. The stress distribution was similar to the results in this paper. Because of the different shape in comparison with the current standard, no conclusion regarding higher or lower stress distributions was drawn concerning the full-overlapped SHS joint with on-top connection. 

Dawod et al. [13] and Soni et al. [14] performed numerical and experimental studies concerning SHS columns under compression loads. No Y-shape was included in these studies. Due to partly eccentric loading, bending was induced in the column. The setup to measure the deflections and stresses was comparable to the laboratory tests in this paper. According to Dawod et al., the slenderness had no influence on the stability because of the small-scale model of the full-overlapped Y-joint. However, the width–thickness ratio was larger for short columns. In future studies, a profile variation will be considered.

## 5. Conclusions

In accordance with to the numerical and experimental studies presented in this paper, the following conclusions are drawn:

1. The inclination angle of the braces has a large influence on the resistance of the welded joints. If the inclination angle increases, the resistance of the joint will decrease significantly. The resistance reduction is around 46% for an increase in inclination of 25°, around 2 percent per one degree; there is reduction when comparing the 20° and 45° models.
2. The behavior of the resistance regarding the different inclination angle systems is not linear. There is a larger effect concerning the extreme angles of 20° and 45°. The results of the 25° and 30° systems are similar. In future studies, more steps regarding the inclination angle in the range of 20° to 45° will be analyzed.
3. The joint made of the squared hollow section profile has a higher resistance compared to the circular hollow section profile joints. The SHS resistance is higher compared to joints made of circular profiles, including the same or smaller profile thicknesses. This conclusion is valid for the joints with the geometrical parameters presented in this paper.
4. By comparing the results of the SHS and CHS models concerning the maximal applicable force, differences occur. The differences are 32 kN between the SHS and CHS and 2.8 mm and 21 kN between the CHS 2.0 mm and CHS 2.8 mm models. These values are averages. These differences of the results are nearly constant independent of the inclination angle.
5. There is good agreement between the numerical and experimental results. Using a refined mesh with solid elements, the differences of the numerical and experimental results are between 9% and 20%. These differences are explainable by small welding imperfections.
6. If the inclination angle increases, the strains at relevant positions will decrease. The numerical and experimental tests show that these relevant positions are generally located at the welding line and at the pipes close to the welding line. In particular, higher strains arise at the flat surface of the pipe, close to the edges of the square hollow section.

These conclusions can help the design engineer optimize the structures, which include welded steel joints with special shapes. By varying the angle of inclination, greater loads can be attributed to the joint. Knowing this, types of structures with lower profiles are achievable, which results in economic benefits for the industry.

## Data Availability

Not applicable.

## References

[1] Tan K.H., Fung T.C., Nguyen M.P. (2013). Structural behavior of CHS T-joints subjected to brace axial compression in fire condition. J. Struct. Eng..

[2] (2010). Eurocode 3, Bemessung und Konstruktion von Stahlbauten—Bemessung von Anschlüssen, 2010–2012.

[3] Wardenier J., Kurobane Y., Packer J.A., Van der Vegte G.J., Zhao X.L. (1991). Design guide for circular hollow section (CHS) connection under predominantly static loading. Construction with Hollow Sections.

[4] Heinemann P., Isopescu D.N., Maxineasa S.G. Numerical case study about three-dimensional CHS joints with overlapped top connection. Proceedings of the CNCM17: XVII National Conference of Metal Constructions.

[5] Heinemann P., Isopescu D.N., Maxineasa S.G. (2021). FEM Analysis for the behavior of two-dimensional CHS joints with asymmetrical Full-Overlapped top-connection. Mater. Today Proc..

[6] Heinemann P., Isopescu D.N., Maxineasa S.G. (2021). Case studies on finite element modelling of welded joints. Bull. Polytech. Inst. Jassy Constr. Archit. Sect..

[7] Heinemann P., Isopescu D.N., Maxineasa S.G. Numerical case studies about two-dimensional SHS joints with symmetrical and asymmetrical top-connection. Proceedings of the 16th International Scientific Conference: Civil Engineering and Building Services (CIBv 2021).

[8] Heinemann P., Isopescu D.-N. (2022). Numerical Case Studies about Two-Dimensional CHS Joints with Symmetrical Full-Overlapped Top-Connection. Materials.

[9] Podkoritovs A., Serdjuks D., Goremikins V., Buka-Vaivade K., Kirsanov M.N. (2020). Behavior of a space inverted triangular steel truss. Balt. J. Road Bridge Eng..

[10] Talabani S.S., Azeez A.T., Barros S.D., Fadhil B.M., Omer H.H. (2020). Effect of Modelling Techniques on the Simulation: Calculating the Stress Concentration Factors in Square Hollow Section T-Joints as a Case Study. ARO-Sci. J. Koya Univ..

[11] Radić I., Markulak D., Mikolin M. (2010). Design and FEM Modelling od steel Truss Girder Joints. Strojarstvo.

[12] Kalač Š., Zejnelagić N., Đuričić Đ., Lučić D. Proposal of analytical expression for determination of load capacity for aluminum square hollow section (SHS) K Joint under chord tension. Proceedings of the 8th International Conference “Civil Engineering—Science and Practice” GNP.

[13] Dawod B.M., Safar S.S. (2021). Experimental and Numerical Investigation on Strength of Eccentrically Loaded Steel Square Hollow Sections. Des. Eng..

[14] Soni V., Chandrakar V.K.S., Tomar P.S. (2018). Experimental Study of Connection for Squared Hollow Beam and Column. Int. J. Sci. Res. Dev..

[15] (2019). Cold Formed Welded Steel Structural Hollow Sections—Part 2: Tolerances, Dimensions and Sectional Properties.

[16] Heinemann P., Isopescu D.N., Maxineasa S.G. (2021). The influence of materials on the behavior of joints with multiple bar connections. IOP Conference Series: Materials Science and Engineering, Proceedings of the 15th International Scientific Conference CIBv—Civil Engineering and Building Services, Brașov, Romania, 5–6 November 2020.

[17] Younise B., Rakin M., Gubeljak N., Međo B., Sedmak A. (2016). Numerical prediction of ductile fracture resistance of welded joint zones. Procedia Struct. Integr..

[18] Konjatić P., Katinić M., Kozak D., Gubeljak N. (2022). Yield Load Solutions for SE(B) Fracture Toughness Specimen with I-Shaped Heterogeneous Weld. Materials.

[19] Saini D.S., Karmakar D., Ray-Chaudhuri S. (2016). A review of stress concentration factors in tubular and non-tubular joints for design of offshore installations. J. Ocean Eng. Sci..

[20] Heinemann P., Isopescu D.N., Maxineasa S.G. (2020). Study on the modelling of crack propagation in the joints of tubular steel elements. Bull. Polytech. Inst. Jassy Constr. Archit. Sect..

[21] Atteya M., Mikkelsen O., Lemu H.G. State-of-the-art of crack propagation modelling in tubular joints. Proceedings of the 2nd Conference of Computational Methods in Offshore Technology and First Conference of Oil and Gas Technology (COTech & OGTech 2019).

[22] Al-Mukhtar M.A. (2011). Fracture Simulation of Welded Joint.

[23] Doncheva E., Medjo B., Rakin M., Sedmak S., Trajanoska B. (2018). Numerical simulation of crack propagation in high-strength low-alloyed welded steel. Procedia Struct. Integr..

[24] Zwick Roell Measuring Machines. https://www.zwickroell.com/.

[25] Teflon Plates, Material: Polytetrafluorethylene (PTFE). https://www.teflon.de/.

[26] Heinemann P., Isopescu D.-N. (2022). Experimental and Numerical Case Studies about Two-Dimensional CHS Joints with a Symmetrical Y-Shape. Materials.

[27] ANSYS Inc. Products 2019 R3^©^ 2006–2019, Software Application. https://www.ansys.com/.

[28] Efthymiou M. (1985). Local Rotational Stiffness of Unstiffened Tubular Joints; KSEPL Report RKER 85.

